# Supplementary figures and images for: Quantifying cumulative phenotypic and genomic evidence for procedural generation of metabolic network reconstructions
Source: PLoS Comput Biol. 2022 Feb 7;18(2):e1009341. doi: 10.1371/journal.pcbi.1009341 (PMC8853471; doi:10.1371/journal.pcbi.1009341)

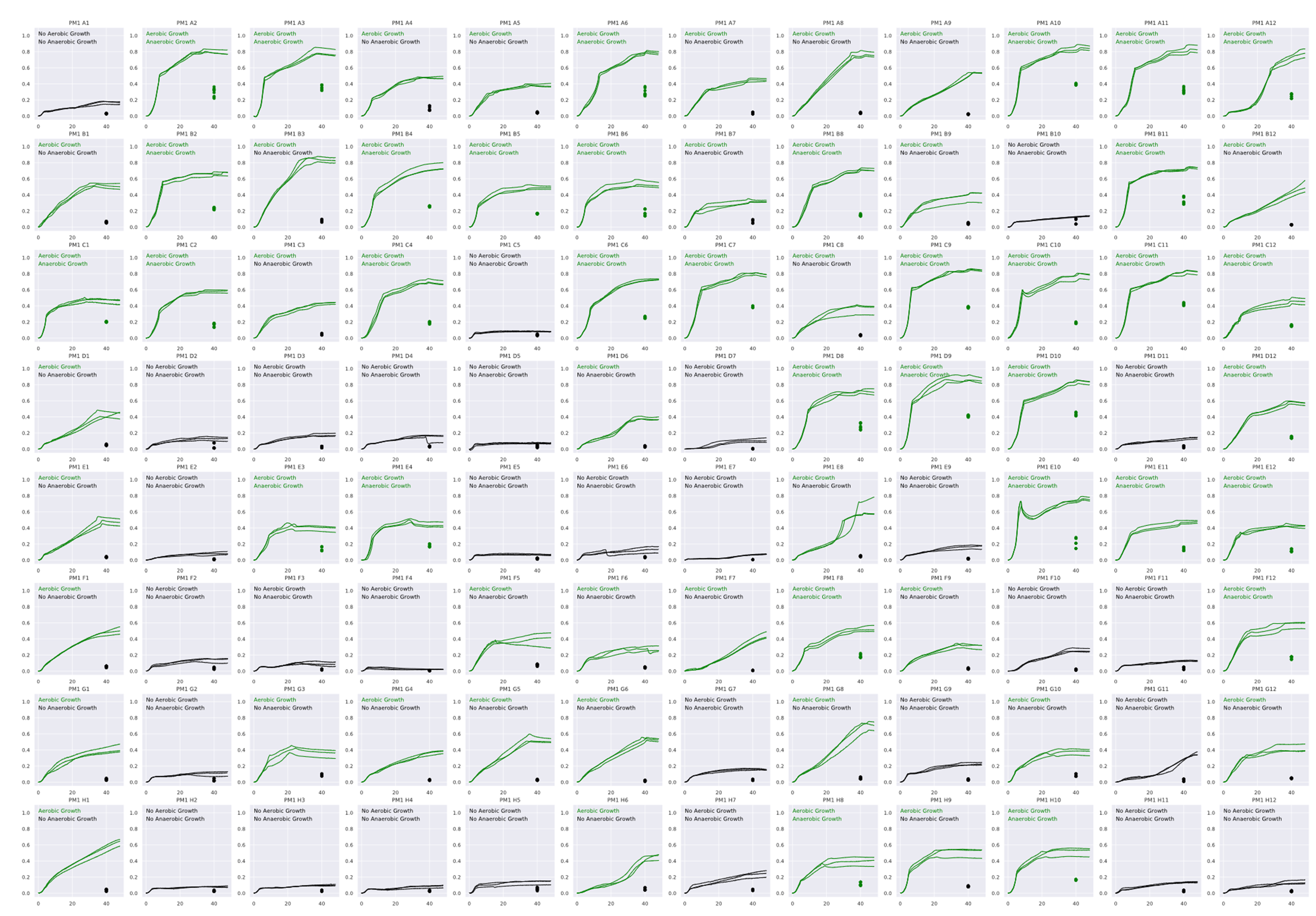

Supplement: S1 Fig — There are 70 aerobic growth conditions and 34 anaerobic growth conditions. The x-axis is time in hours and the y-axis is OD measured at 600 nanometers. (TIF) [file pcbi.1009341.s001.tif]

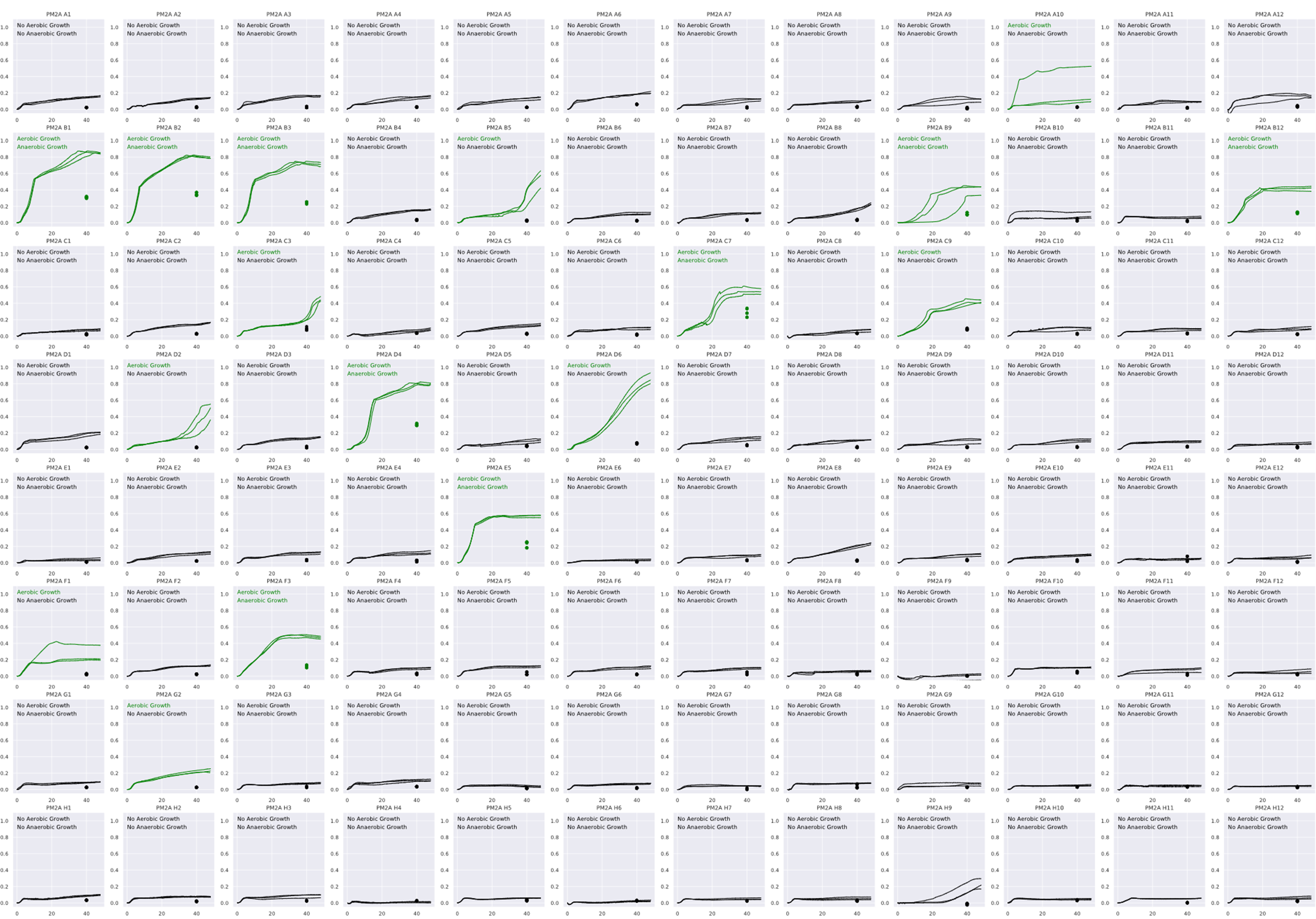

Supplement: S2 Fig — There are 22 aerobic growth conditions and 9 anaerobic growth conditions. The x-axis is time in hours and the y-axis is OD measured at 600 nanometers. (TIF) [file pcbi.1009341.s002.tif]
