# Supplementary material for: Quantifying cumulative phenotypic and genomic evidence for procedural generation of metabolic network reconstructions
Source: PLoS Comput Biol. 2022 Feb 7;18(2):e1009341. doi: 10.1371/journal.pcbi.1009341 (PMC8853471; doi:10.1371/journal.pcbi.1009341)
Supplement: S1 Table — Anaerobic data for K-12 growth in Biolog plate PM2A was not available on EcoCyc. (DOCX) [file pcbi.1009341.s003.docx]

| **Index** | **Metabolite Name** | **Biolog Plate and Well #** | **Culture Environment** | ***E. coli* K-12 Growth** | ***E. coli* Nissle Growth** |
| --- | --- | --- | --- | --- | --- |
| 1 | Dulcitol | PM1 – A12 | Aerobic | No Growth | Growth |
| 2 | L-Glutamic acid | PM1 – B12 | Aerobic | No Growth | Growth |
| 3 | Glucuronamide | PM1 – H7 | Aerobic | No Growth | Growth |
| 4 | N-Acetyl-D- Galactosamine | PM2A – B1 | Aerobic | No Growth | Growth |
| 5 | D-Raffinose | PM2A – D1 | Aerobic | No Growth | Growth |
| 6 | Salicin | PM2A – D2 | Aerobic | No Growth | Growth |
| 7 | L-Alaninamide | PM2A – G2 | Aerobic | No Growth | Growth |
| 8 | D-Lactitol | PM2A – C3 | Aerobic | No Growth | Growth |
| 9 | L-Sorbose | PM2A – D4 | Aerobic | No Growth | Growth |
| 10 | D-Arabinose | PM2A – B5 | Aerobic | No Growth | Growth |
| 11 | D-Tagatose | PM2A – D6 | Aerobic | No Growth | Growth |
| 12 | Arbutin | PM2A – B8 | Aerobic | No Growth | Growth |
| 13 | β- Hydroxybutyric acid | PM2A – E8 | Aerobic | No Growth | Growth |
| 14 | 2-Deoxy- D-Ribose | PM2A – B9 | Aerobic | No Growth | Growth |
| 15 | Laminarin | PM2A – A10 | Aerobic | No Growth | Growth |
| 16 | M-Tartaric acid | PM1 – E2 | Aerobic | Growth | No Growth |
| 17 | D-Fructose- 6-Phosphate | PM1 – E4 | Aerobic | Growth | No Growth |
| 18 | Acetoacetic acid | PM1 – G7 | Aerobic | Growth | No Growth |
| 19 | α- Ketobutyric acid | PM1 – D7 | Aerobic | Growth | No Growth |
| 20 | α- Hydroxybutyric acid | PM1 – E7 | Aerobic | Growth | No Growth |
| 21 | 5-Keto-D- Gluconic acid | PM2 – E12 | Aerobic | Growth | No Growth |
| 22 | D-Serine | PM1 – B1 | Anaerobic | Growth | No Growth |
| 23 | D-Saccharic acid | PM1 – A4 | Anaerobic | Growth | No Growth |
| 24 | L-Galactonic acid-γ- Lactone | PM1 – H9 | Anaerobic | Growth | No Growth |
| 25 | Methylpyruvate | PM1 – G10 | Anaerobic | Growth | No Growth |
