# Supplementary material for: Quantifying cumulative phenotypic and genomic evidence for procedural generation of metabolic network reconstructions
Source: PLoS Comput Biol. 2022 Feb 7;18(2):e1009341. doi: 10.1371/journal.pcbi.1009341 (PMC8853471; doi:10.1371/journal.pcbi.1009341)
Supplement: Attachment — Submitted filename: Moutinho_Papin_Response_to_Reviewers.pdf [file pcbi.1009341.s004.pdf]

We appreciate the thoughtful consideration of the reviewers and editors. Below we address each of the comments from the reviewers. Original reviewer comment in **black** and our responses in **blue**.

Comments to the Authors:
Please note here if the review is uploaded as an attachment.

Reviewer #1: In the present paper Thomas J. Moutinho Jr. and colleagues presented a novel method for quantifying the combined genomic, biochemical, and phenotypic evidence for all the biochemical reaction during automated genome-scale metabolic network reconstruction. Authors reported that the method generates accurate GENREs supported by a quantitative metric describing the cumulative evidence for each biochemical reaction included in the network. Additionally, they built a new CANYUN GENRE for E. coli Nissle using phenotypic data specifically collected for the project. The paper is clear and well-written, the cited literature covers in general the previously published studies related to the topic, despite the addition of some other references was suggested (see details below).

We appreciate the review of our work.

Detailed comments to the authors.
General comment. The approach described here can be useful to improve the annotation process in general and not only when propedeutic to the generation of a GENRE. Authors could consider to include a general comment suggesting similar improvements for the software used for microbial genome annotation (here annotation refers to functional assignment).

This is an interesting idea. We have added text to the conclusion to incorporate this concept with our existing comments about how this method may be valuable for expanding the sequence-to-reaction dataset that is used to determine the reaction bitscores. (Lines: 447-449)

General comment. Authors tested their approach in a microbial species (E. coli) which is well studied, easy to annotate and easy to grow. I am wondering if the present approach can also be applied to other microbial species which are taxonomically distant from other well studied microbes and difficult to cultivate. Obviously, limitations in growing microbes cause limitations in obtaining phenotipic data. Authors can provide a short explanation regarding the limitations of the proposed approach.

This is a valuable point; we have added a paragraph with several limitations in the Conclusion (Lines: 429-440).

General comment. Consistency is important to ensure the theoretical soundness of models (e.g., mass and charge balance, absence of energy-generating cycles) and

model validation is important to ensure that a model describes the biological reality of the organism it is representing. Consistency should be systematically curated and assessed, preferably passing the standardized MEMOTE test suite (https://memote.io/).

We utilized MEMOTE to determine that the CANYUNs models we generated for K-12 and Nissle are 100% Stoichiometric Consistent. The models are saved in the repo under the names: 'k12_canyun.json' and 'nissle_canyun.json'. This result required a few minor adjustments to metabolite formulas to ensure mass balance in all of the reactions, resulting in minor changes to exact reactions that are in each model. These changes can be seen in Figures 3, 4, 6, 7, and 8. Ultimately, none of the conclusions or results have changed as a result of these minor adjustments.

General comment. The github page of the tool is not weel structured, a readme and a manual page are missing.

Thank you for the suggestion, the github repo has been restructured with a ReadMe file for easier utilization of the CANYUN functions.

General comment. To date 16/08/2021 the _init.py_ file in Github is empty; probably this is ok, but please verify.

Thank you for the notification. We have added to and verified the _init.py_ file.

General comment. The data utilized for validating CANYUN on E. coli K-12 are only briefly mentioned and should be described more in detail with related web links for clarity and full reproducibility.

Additional information about the *E. coli* K-12 phenotypic growth data has been added to the Methods with associated links. (Line 528)

General comment. The methods should provide more details over the simulation parameters, such as metabolite exchange bounds, non-null growth threshold, etc. to allow full reproducibility.

We have added these details to the methods. (Lines 484-499)

General comment. More precise inputs, data requirements, and limitations should be provided for a more accessible utilization by other researchers. For example, while it is clear that CANYUN was developed and validated with binary growth/no-growth phenotypic screens, a potential user might wonder if other phenotypic data could be used. If only binary growth/no-growth datasets are supported by CANYUN, this should be clearly discussed along with its other limitations.

We have added this detail to the Discussion. (Lines 429 – 440)

Line 7. Authors can consider to include recently reported collections of GENREs such

as those obtained in (doi: 10.1038/s41559-020-01353-4) and in (https://doi.org/10.1016/j.ymben.2020.08.013).

We have added these references to the Introduction.

Line 65. Please consider to include more references such as (doi: 10.1093/bioinformatics/btab324).

We have added this reference to the Introduction.

Line 99. The authors selected CarveMe as a tool to generate the draft GENRE; did authors consider other tools as well? (e.g. RAVEN, gapseq, Merlin)? Why did authors specifically selected CarveMe?

We utilized CarveMe because it allows for the most direct comparison with CANYUNs. The reactions are represented by the same stoichiometries and nomenclature as iML1515, thus providing a clear and direct comparison. Additionally, CarveMe is built upon a well-documented sequence-to-reaction reference dataset that allows for explicit tracking and maintenance of all input data required for building a metabolic network.

Line 110; lines 151-156. Authors used BLASTp to align the genome of the target organism with reference sequences in the CarveMe sequence-to-reaction dataset. The sequence alignment bitscores for E. coli K-12 genes and the CarveMe dataset were used to generate reaction bitscores using previously published methods. However, the simple use of BLASTp can limit the efficiency of the process, did authors consider the possibility to use other tools such as for example HMM search?

The reviewer has identified a useful idea to increase the speed of genome alignment with the gene-to-reaction reference dataset. The authors did not explore the use of methods faster and more efficient than BLASTp due to the small size of the reference dataset, limiting the overall utility of reducing the time it takes to generate an alignment. However, with larger reference datasets an HMM search would definitely save time. We have added this to the discussion (Line 439).

Lines 421 and following. The generation of models for poorly studied microorganisms is not explained exhaustively, in particular the importance of obtaining biochemical data and to perform growth tests should be clarified.

This key limitation of CANYUNs has been added to the Discussion. (Lines 429 – 440)

Line 470 and following. Information about the solver(s) used has not been provided and should be included in Materials and Methods.

We have added text to the methods to discuss the solver utilized in this study. We used GLPK, the default solver for Cobrapy. We added this detail to the Methods.

Lines 328 and following. This last part is not very easy to understand. I would expect to see some results reporting at what level the implementation of the proposed approach and the generation of the CANYUN GENRE can recapitulate in a more reliable way the results obtained from biolog experiments.

We have adjusted the text (now around line 337) in this section to add clarity. We also show in Figure 8 the accuracy of the *E. coli* Nissle CANYUN model to be 92% in recapitulating the phenotypic growth data we generated.

Reviewer #2: Moutinho and colleagues describe a new computational framework for automatic generation of genome-scale reconstruction networks that improves upon pre-existing methods by integrating genomic, biochemical, and phenotypic (i.e. growth conditions) to more accurately represent organism-specific metabolism by constraint-based modeling. There is a pressing need in genome-scale metabolic modeling to eliminate manual curation of reconstructed networks due the sheer number of bacterial species and strains implicated in microbiome applications; thus the impact of this paper is potentially high. I particularly like how the proposed model curation methodology leverages the wealth of phenotypic data available to add confidence in a metabolic network based upon ensemble performance simulating the experimental conditions. The authors compare “ground truth” of a manually curated model to a model generated by their pipeline and evaluate accuracy with pre-existing computational tools as a means of benchmarking their results. They test their computational tools on a new E. coli Nissle network by generating new experimental Biolog growth data for the purposes of this study.

We appreciate the reviewers’ encouragement and critiques which have helped us to improve the manuscript.

I find only minor issues with the manuscript in its current form which, if addressed, will increase the readership and usage of their CANYUNs code.

1. Understandably the authors are focused on human health applications with the K-12 strain, however the CANYUNs applications could be easily extended to environmental microbiota (water sources, soil samples) and the paper could be generalized slightly in the introduction to extend potential adoption of the pipeline.

Thanks for the suggestion; we have adjusted the Introduction to reflect this comment.

2. Along the same lines, there is no associated documentation in the github README file for actual implementation of the files deposited. More description for the user here would be helpful.

Great suggestion; we have added instructions to the README file on Github.

3. The description of calculated reaction bitscores, which is central to the generation of

the CANYUNs, confidence in reactions, and data guided FBA interpretation of the models, seems incomplete. The authors cite reference 10 on the bitscore calculation. The original CARVEME algorithm yields a log-normal distribution in scoring. The distribution of reaction bitscores used to define thresholds in Figures 1 and 2 do not display this feature, and there is not discussion on the implications of this for the weights applied in the dgFBA generation.

We did not normalize the bitscores generated using the method described in reference 10. All other steps remain identical to the published method. We have clarified this point in the Methods. (Line 504)

4. The Certainty Value is a useful construct for quantifying confidence in a reaction by the frequency of flux occurring across all experimental conditions, thereby leveraging numerous media growth condition datasets. While this may be defined as a simple ratio, it should be explicitly included in the CANYUNs pipeline description in the Methods section.

We have added this point to the Methods. (Lines 507-514)

Reviewer #3: The manuscript proposes a new framework for generating genome-scale metabolic network reconstructions. Instead of making a draft metabolic network and subsequently performing a gap-filling, the authors propose to simultaneously integrate genome annotation and phenotypic growth data. The authors demonstrate through a well-chosen set of comparative studies the usefulness of such a holistic approach. While the manuscript is well-written and the text is, for the most part, clear, some methodological passages need additional clarification and discussion. My specific comments follow.

Comments:

1. A similar optimization problem of maximizing the number of high genomic evidence reaction and minimizing low evidence reactions already exists in CarveMe. Though the authors are apparently aware of this work, they do not discuss what are the advantages and disadvantages of the LP dgFBA formulation compared to the MILP CarveMe formulation. In the discussion, it would be important to place both optimization problems in the context of their enveloping methods (CANYUN and CarveMe). The readers should be able to assess clearly the contribution of this work and this discussion would be a key part in this process.

We have added a section to the Methods to discuss dgFBA, LPs, and MILPs to address this point. (Lines 484 – 499)

2. Whereas this might be obvious to some of the readers, the elements in the reaction flux vector, v, in dgFBA can take only positive values (otherwise, the reactions in the forward direction with the high genetic annotation evidence and the reactions in the reverse direction with the low evidence would have the same weight in the objective).

The authors should state this explicitly in their problem formulation (Fig. 2) to avoid confusion.
In this line, since the directionality of the reactions directly influences the criterion function, one has to explore all possible combinations of forward/reverse reactions to obtain the optimal objective value. Considering that in the studied network there are 45 reactions that can operate in either of the directions (line 221), it seems that one has to analyze 2^45 cases, which is computationally intractable. One can reduce this number by performing a flux coupling analysis and assign certain directionalities from the literature, however, the number of the cases can still be daunting.
It seems that this issue of the proposed optimization problem is completely neglected in the manuscript. This issue is not new and specific only to this work and it hampers some other methods in the field of constraint-based modeling, however, it has to be acknowledged and discussed how to alleviate it.

Each reversible reaction is represented as two opposite reactions that each allow flux in the positive direction. In some cases, these two reactions both carry flux and therefore the one flux value is subtracted from the other to calculate the final reported flux for the reversible reaction (based on the original orientation of the reaction). This process allowed us to avoid analyzing any combinations of irreversible reactions to determine the directionality of the flux through a reaction in a given growth condition. We have added clarification on this point to the Methods (Lines 484-499, 509-516).

3. Lines 212-214, the authors state “the directionality of each flux value is used to specifically determine the cumulative evidence for each reaction specific to direction”. The authors did not specify precisely how this is done. In the light of my remark 2, when testing the cumulative evidence of one reaction, the chosen directionality of other reactions will influence the results. This should be discussed and clarified.

We have added this detail to the Methods as well. When calculating the Certainty Values for each reaction, there is one value for the forward direction and one for the reverse direction. Since flux can only move in one direction in each growth condition, the sum of the two certainty values for a given reaction will never exceed 1. If a reversible reaction in the universal model only carries flux in the forward direction and never in the reverse direction, it will receive a certainty value for only the forward direction and thus will be only represented as a unidirectional reaction in the CANYUN model. We have added clarity on this point to the Methods (Lines 484-499, 509-516).

4. Though dgFBA identifies a set of reactions that can carry flux given certain phenotypic data, I am not sure that the authors can talk about “flux distributions” of dgFBA in the sense that dgFBA solutions would probably not have flux values corresponding to the actual intracellular fluxes. For example, in pFBA, the solutions would likely correlate to the metabolic states with minimal dissipation. In contrast, in dgFBA, the reactions with weights close to zero can take as high values as the steady-state constraint (S*v=0) allows.

This point is absolutely true; we have added to the text around line 178 for clarification. We do not intend for the term flux distribution to convey expected biological flux.

5. The authors curate universal network by manually removing reactions that were contributing to free-mass generation. Once the proposed method identified the reactions taking part in the free-mass generation, it is not clearly explained whether all reactions that are taking part in the free-mass generation are removed or just a few of them. If only a part of reactions is removed, how the authors choose which reactions to remove and what are the recommendations to do this? Otherwise, if all reactions are removed, why the authors emphasize that they are removed “manually”? Further down in the results section, how many reactions were removed? Were the removed reactions originating from iML1515, the CarveME network, or both? Moreover, it is reasonable to assume that these reactions, if appearing in iML1515 or in the created CarveME model would affect the essentiality analysis. Can you comment on this?

Each mass generating loop was inspected to determine the intervention that would allow for minimal adjustments to be made in order to fix the issue. Unfortunately, this type of curation required manual intervention and human judgement to complete. We utilized reactions that originated from iML1515 and removed reactions that were clearly incomplete or completely mass-imbalanced from the CarveMe universal model. We also adjusted one reaction ('BTS_1') that needed hydrogen ions removed due to our decision to default to the metabolite formulas from the iML1515 model. The metabolites in the original CarveMe universal model are represented with multiple formulas each, which is incompatible with MEMOTE. There were 178 reactions removed from the universal model to allow for the elimination of mass generating loops. All reactions removed can be found in the file named ‘trouble_reaction_ids.pickle’ in the github repository. We created the CarveMe models and the CANYUN models using the same set of universal reactions. We believe is it highly likely that there are reactions that remain in our universal model that could cause issues with generating CANYUN models, just as we saw with the presence of RuBisCo in the K-12 model. However, that is the intention of generating a draft CANYUNs model and providing a solution to quickly identify the reactions that may be removed to provide a net benefit to the overall accuracy of the model.

6. In general, for testing this and other possible issues in the genome-scale models, the MEMOTE method by C. Lieven et al (Nat Biotech 38, pp 272–276 (2020)) is the community standard. Were the obtained models tested using this tool? Would MEMOTE identify the issues with the free-mass generation and if yes, how the proposed method is different than the one used in MEMOTE?

We utilized MEMOTE to determine that the CANYUNs models we generated for K-12 and Nissle are 100% Stoichiometric Consistent. The models are saved in the repo under the names: ‘k12_canyun.json’ and ‘nissle_canyun.json’. This result required a few minor adjustments to metabolite formulas to ensure mass balance in all of the reactions,

resulting in minor changes to exact reactions that are in each model. These changes can be seen in Figures 3, 4, 6, 7, and 8. Ultimately, none of the conclusions or results have changed as a result of these minor adjustments.

MEMOTE relies heavily on each reaction to be mass-balanced in order to determine Stoichiometric Consistency. The method that we utilized allowed for some flexibility in the metabolite formulas, but still allows for identification of any metabolites being freely generated. With the use of MEMOTE, we are now confident that our resulting CANYUNs models have Stoichiometric Consistency.

7. Concerning the confusion matrices presented in Figure 5, the authors use as the performance measure the accuracy. Considering that the analyzed sets are not symmetric, a more balanced measure for this would be the Matthews correlation coefficient.

We have added the Matthews Correlation Coefficient to all of the accuracy calculations. We chose to include it in addition to the overall accuracy, in the case a reader doesn't have a good frame of reference for evaluating MCC values.

8. Lines 389-391, the authors state that there is no need for further thermodynamic curation of the resulting GENREs – thermodynamics can also give us information about thermodynamically-feasible directionality of reactions in the network, which can reduce the number of reactions operating in both forward and reverse direction.

We have adjusted the statement to disconnect it from consideration of thermodynamics. You are absolutely correct that thermodynamics provides valuable and essential information about reaction directionality. Some thermodynamic information has been accounted for in our universal model based on the existence of unidirectional reactions. However, this is an area in which there is a need for additional research and curation.

9. It would be good that the authors discuss why the number of false negatives from 3 in the initial CANYUN model (Fig. 4d) increased to 10 in the final model (Fig. 5d).
10. Figure 1, it seems that the network has more of the exchange metabolites than the exchange reactions. Can you explain that?

There are four metabolites that did not get exchange reactions because they were not present in the original universal models used for building our universal model and they are also not present in any media conditions. The metabolites are chromate, l-cystine, fluoride, and pseudouridine. For the work here, we did not see a reason to add these exchanges to the universal model. However, it would be easy for any future users to identify and add these exchange reactions to the universal model as desired.

In the caption for the panel e), the authors should mention what is the orange dashed line and explain the bar left to that line (1/3 of reactions with zero bitscore).

We added this point to the caption; thank you for identifying that issue.

11. It is not entirely clear to me what was the purpose of showing the distribution of reaction weights in the forward and reverse direction (Figure 2c and d)? Besides, these figures were not referred to in the main manuscript.

We had added text to the results referencing these panels. We believe these panels to be valuable information for a reader looking to better understand how the transformation function utilizes the bitscores to determine the reaction weights. (Lines 156-158)

12. Figure 4a, b, and, c what is the information that the authors wanted to convey and how to read these panels?

These panels provide a visual representation of the Certainty Values associated with each reaction. We believe these panels provide valuable information for a reader who is looking to gain a better understanding of what the distribution of values may look like for a CANYUNs model. In panels A and B you can see the reactions that receive only one certainty value, either in the forward or reverse direction. For both of these, there is a core set of reactions that carry flux in nearly all of the growth conditions. Whereas, there is also a large set of reactions that only carry flux in 1-2 growth conditions. In panel C, we are showing the reaction that receives CVs in both directions. The reactions with CVs summing closer to 1 carry flux in nearly all of the growth conditions.

13. Line 459, cite the Diamond method.

We had added this reference.
